# Bioreactor development for skeletal muscle hypertrophy and atrophy by manipulating uniaxial cyclic strain: proof of concept

**DOI:** 10.1038/s41526-023-00320-0

**Published:** 2024-06-11

**Authors:** Khaled Y. Kamal, Mariam Atef Othman, Joo-Hyun Kim, John M. Lawler

**Affiliations:** 1 Redox Biology & Cell Signaling Laboratory, Department of Health and Kinesiology, Graduate Faculty of Nutrition, Texas A&M University, College Station, TX USA; 2 Department of Nutrition, Texas A&M University, College Station, TX USA

**Keywords:** Cell biology, Physiology

## Abstract

Skeletal muscles overcome terrestrial, gravitational loading by producing tensile forces that produce movement through joint rotation. Conversely, the microgravity of spaceflight reduces tensile loads in working skeletal muscles, causing an adaptive muscle atrophy. Unfortunately, the design of stable, physiological bioreactors to model skeletal muscle tensile loading during spaceflight experiments remains challenging. Here, we tested a bioreactor that uses initiation and cessation of cyclic, tensile strain to induce hypertrophy and atrophy, respectively, in murine lineage (C2C12) skeletal muscle myotubes. Uniaxial cyclic stretch of myotubes was conducted using a StrexCell® (STB-1400) stepper motor system (0.75 Hz, 12% strain, 60 min day^-1). Myotube groups were assigned as follows: (a) quiescent over 2- or (b) 5-day (no stretch), (c) experienced 2-days (2dHY) or (d) 5-days (5dHY) of cyclic stretch, or (e) 2-days of cyclic stretch followed by a 3-day cessation of stretch (3dAT). Using ß-sarcoglycan as a sarcolemmal marker, mean myotube diameter increased significantly following 2dAT (51%) and 5dAT (94%) vs. matched controls. The hypertrophic, anabolic markers talin and Akt phosphorylation (Thr308) were elevated with 2dHY but not in 3dAT myotubes. Inflammatory, catabolic markers IL-1ß, IL6, and NF-kappaB p65 subunit were significantly higher in the 3dAT group vs. all other groups. The ratio of phosphorylated FoxO3a/total FoxO3a was significantly lower in 3dAT than in the 2dHY group, consistent with elevated catabolic signaling during unloading. In summary, we demonstrated proof-of-concept for a spaceflight research bioreactor, using uniaxial cyclic stretch to produce myotube hypertrophy with increased tensile loading, and myotube atrophy with subsequent cessation of stretch.

## Introduction

Mitigating the adverse effects of spaceflight requires a better mechanistic understanding of the profound challenges that are imposed upon astronauts, including the upcoming missions to Mars and the moon. Extended spaceflight missions that travel beyond the cocoon of the Earth’s magnetosphere subject astronauts to microgravity and outer space radiation. Risks inherent to the mechanical unloading of microgravity include degeneration of musculoskeletal tissue and the cardiovascular system. The microgravity of spaceflight indeed causes substantial wasting of skeletal muscle and weakness^1–4^. Mounting evidence indicates that altered mechanotransduction, oxidative stress, and impaired stress response contribute to mechanical unloading-induced atrophy and remodeling of musculoskeletal tissue^4–8^. Therefore, the identification of mechanosensing pathways during the unloading of spaceflight, and thus the effect on musculoskeletal morphology and contractile function, continues as one of NASA’s long-term mission objectives.

Skeletal muscles are dynamic tissues that drive body movement, protect joints and bones, serve as the primary glucose sink, and are central to systemic energy metabolism. Skeletal muscles adapt readily to changes in various stimuli, including changes in loading^9,10^. Indeed, skeletal muscles remodel their morphology in response to changes in the mechanical, nutrient, and energy-sensing environments^11–15^. Load-bearing skeletal muscles adapt to changes in the mechanical environment, allowing mature muscle fibers to hypertrophy in response to increased loading and atrophy when presented with decreased loading. For example, when humans participate in resistance exercise, skeletal muscles can undergo increased mass through hypertrophy of muscle fibers^16,17^. In response to mechanical unloading that occurs during the microgravity of spaceflight, chronic bedrest, or casting, skeletal muscles adapt rapidly by degrading contractile protein into constitutive amino acids, suppressing protein synthesis, and thus reducing muscle fiber cross-sectional area^4,6^. A common teleological argument for skeletal muscle atrophy as a biological response to unloading is that extreme disuse would accompany a significant injury or disease, both of which require an increased demand for amino acids and protein^18–20^. However, the skeletal muscle wasting or atrophy that occurs with microgravity^21^, disuse^22^, cancer^23^, and aging^24^ compromises physical function and systemic health.

Rotating wall vessel bioreactors are frequently utilized in tissue engineering and cell culture research to simulate spaceflight microgravity conditions^25–27^. These bioreactors produce a low-shear, three-dimensional culture environment that mimics certain aspects of weightlessness^27^. However, they are not suitable for inducing tensile mechanical loading in skeletal muscle fibers experience during exercise in spaceflight. Although tumbler bioreactors create a low-shear environment through rotation, they largely produce compression rather than tension on skeletal muscle fibers. Therefore, tumbler or centrifugal fail to accurately reproduce the changes in tensile mechanical forces encountered by skeletal muscles during weightlessness and exercise.

Skeletal muscles produce and transfer tensile loads to attached tendons and bones by two mechanisms: (1) in series through muscle fibers and (2) in parallel through transverse loading of z-discs and linked costamere interaction with surrounding layers of connective tissue (e.g., endomysium, perimysium)^28^. Skeletal muscles are organized as multinucleated myofibers, whose function is to generate length and velocity-dependent forces for movement and stability. Skeletal muscle contractions and outside resistance produce net tension down a skeletal muscle’s long axis. Indeed, skeletal muscles respond to dyanamic alterations in mechanical loading by adapting muscle mass, fiber cross-sectional area^29,30^, and shifting the % of slow-twitch fibers^31,32^. Unloading induced skeletal muscle atrophy is believed to be a function of both elevated protein degradation coupled with decreased protein anabolism^8^. In addition, there is an increase in connective tissue in unloaded skeletal muscle as well^33^. Thus, maintaining mechanical loading, such as tension and stretch, is crucial for muscle health and function. Therefore, the development of a bioreactor for experimental use of skeletal muscle cells (i.e., myotubes) to simulate spaceflight or exercise during spaceflight should impose similar uniaxial tensile loading or stretch, that cannot be currently reproduced with rotating wall vessels bioreactors.

Ergo, to study the physiological responses of skeletal muscle fibers to tensile loading, myoblasts were seeded and differentiated into myotubes on an laminin-coated elastic membrane, which was then stretched to observe the cellular responses^34^. Based upon our findings we propose a microgravity bioreactor for the cellular stretch that is an analog of the FlexCell® system, which uses a vacuum instead to stretch the silastic membrane, eliciting radial loading^35^. Instead, we employed a StrexCell® system fitted that includes a stepper motor to produce true uniaxial, cyclic stretch while eliciting tensile loading on myoblasts or myotubes. Therefore, the purpose of this study is to use cyclic stretch and cessation protocols with the StrexCell system on skeletal muscle myotubes^36,37^ as a potential bioreactor for microgravity and re-entry into gravitational environments.

## Results

### Myotube morphology in response to cyclic stretch, cessation of daily stretch

**Fig. 1:**
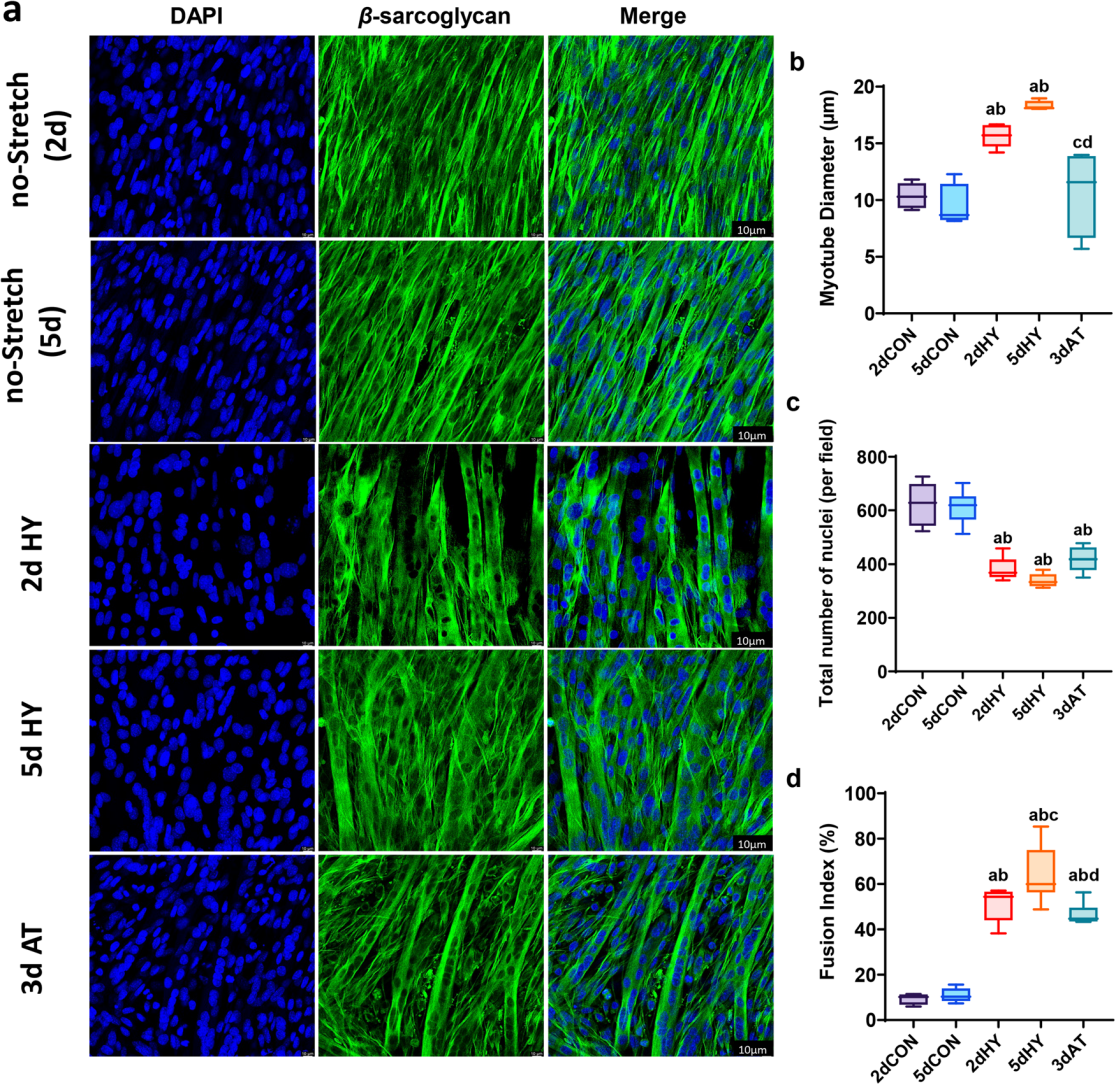
Effect of cyclic stretching and cessation of stretching periods on myotubes size. **a** Confocal fluorescence staining images for C2C12 myotubes. Immunofluorescence staining for ß-sarcoglycan in green was used as a membrane marker with DAPI staining in Blue. **b** Quantification of the Confocal fluorescence staining images for myotubes diameter. **c** Total number of nuclei based on the DAPI staining quantifications. **d** Fusion index of muscle myotubes. Myotubes were divided into the following groups (*n* = 4/group): 2 days quiescent control (no stretch (2d), 5 days quiescent control (no stretch (5d), 2 days cyclic stretch (2dHY), 5 days cyclic stretch (5dHY), and 2-days of cyclic stretch followed by a 3-day cessation of stretch (3dAT). (**a**) = statistically significant difference from no-stretch (2d); (**b**) = significantly different than no-stretch (5d); (**c**) = significantly different than 2dHY; (**d**) = significantly different than 5dHY. (*p* ≤ 0.05) Values are presented as Box plots ±SD. Scale bar = 10 µm. The boxplots illustrate the interquartile range (25th–75th percentiles) displayed with whiskers spanning from the 10th to the 90th percentiles. The median of the data is indicated by the central line.

To study the effects of cyclic stretch and cessation of stretch on skeletal muscle myotube morphology and size, we used C2C12 mouse lineage myoblasts to generate myotubes, and mounted on laminin. *β*-sarcoglycan immunofluorescence positive staining was used as a membrane marker to calculate the myotube diameter as a marker of hypertrophy and atrophy (Fig. 1). Confocal immunofluorescence quantification revealed a significant increase of 51% and 94% in mean myotube diameter following 2 and 5 days of a cyclic stretch, compared to the 2-day and 5-day quiescent controls, respectively (Fig. 1b). When myotubes underwent three days without cyclic stretch in myotubes, following 2 days of cyclic stretch, then myotube diameter was 32% lower compared to the loaded, stretched myotubes (Fig. 1b). Quantification of the total nuclei population per field revealed a significant reduction as a result of 2 and 5 days of cyclic stretching compared to the controls (Fig. 1c), indicating an increase in fiber size while the nuclear number was relatively static. The fusion index was found to be increased significantly by the 2, and 5 days stretching protocol compared with quiescent controls (Fig. 1d). However, three days of cessation of daily bouts of cyclic stretch (3dAT), did not alter the fusion index compared to the 2 days cyclic stretching.

### Functional protein turnover marker with cyclic stretching and cessation of stretch

**Fig. 2:**
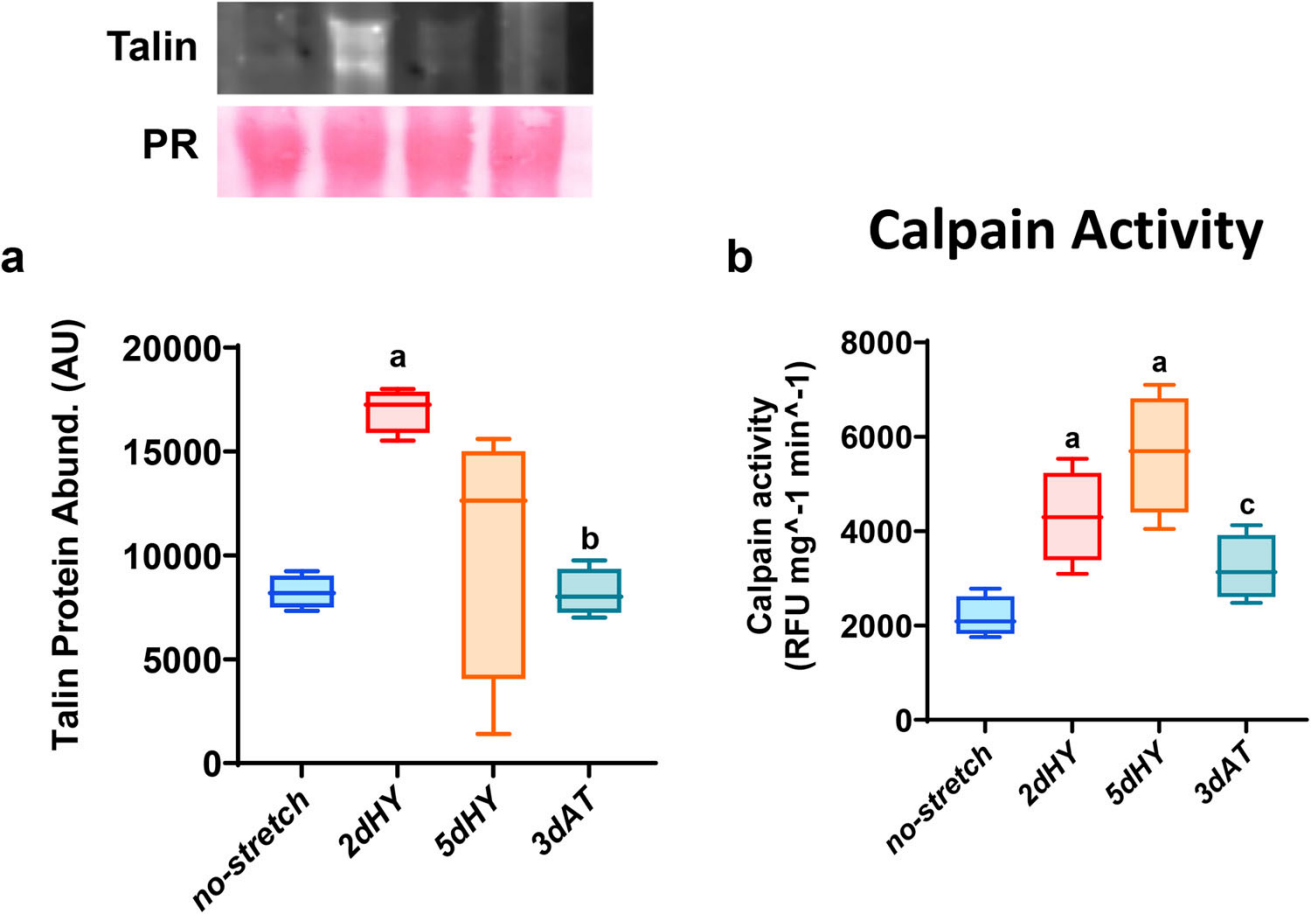
Markers of protein turnover: Talin protein levels and calpain activity assay. **a** Protein abundance for talin protein levels. Protein abundance quantification was determined using Western immunoblotting with Ponceau stain as a protein loading control and quantified using ImageJ for talin. **b** Calpain Activity is expressed as fluorogenic values (RFU/mg/min). Myotubes were divided into the following groups (*n* = 4/group): 2 days quiescent control (no stretch (2d), 2 days cyclic stretch (2dHY), 5 days cyclic stretch (5dHY), and 2-days of cyclic stretch followed by a 3-day cessation of stretch (3dAT). (**a**) = statistically significant difference from no-stretch (2d); (**b**) = significantly different than 2dHY; (**c**) = significantly different than 5dHY. (*p* ≤ 0.05) Values are presented as Box plots ±SD. The boxplots illustrate the interquartile range (25th–75th percentiles) displayed with whiskers spanning from the 10th to the 90th percentiles. The median of the data is indicated by the central line.

We then determined the effects of (1) uniaxial cyclic stretching and (2) release from or cessation of daily bouts of cyclic stretch on markers of protein synthesis and proteolysis commonly used in muscle atrophy and hypertrophy studies^35,38,39^. Protein abundance of the focal adhesion complex and cytoskeletal protein talin was chosen as a muscle hypertrophy marker, while the activity of calpains was chosen as a proteolytic marker (Fig. 2). Our findings revealed that both 2 and 5 days of cyclic stretching significantly elevated the levels of talin protein compared to the control levels (Fig. 2a). There was no statistically significant difference between 2 and 5 days of stretch on talin protein levels.

However, three days of cessation of daily bouts of cyclic stretch (3dAT) resulted in a significantly lower level of talin than 2 and 5 days of daily cyclic stretch. Talin protein levels were not statistically different from quiescent myotubes that experienced no daily stress and strain due to stretch.

In contrast, calpain activity that was measured by fluorescence revealed a significant increase in activity in the 2dHY and 5dHY groups, indicating that daily bouts of cyclic stretch elevated calpain levels and presumably remodeling (Fig. 2b). Myotubes that completed stretch cessation myotubes displayed calpain levels that were not statistically different than the no-stretch and 2dHY groups. Calpain activity was significantly lower in the 3dAT group vs. 5dHY myotubes.

### Akt and Foxo3a signaling is altered by cyclic stretch and removal of cyclic stretch

**Fig. 3:**
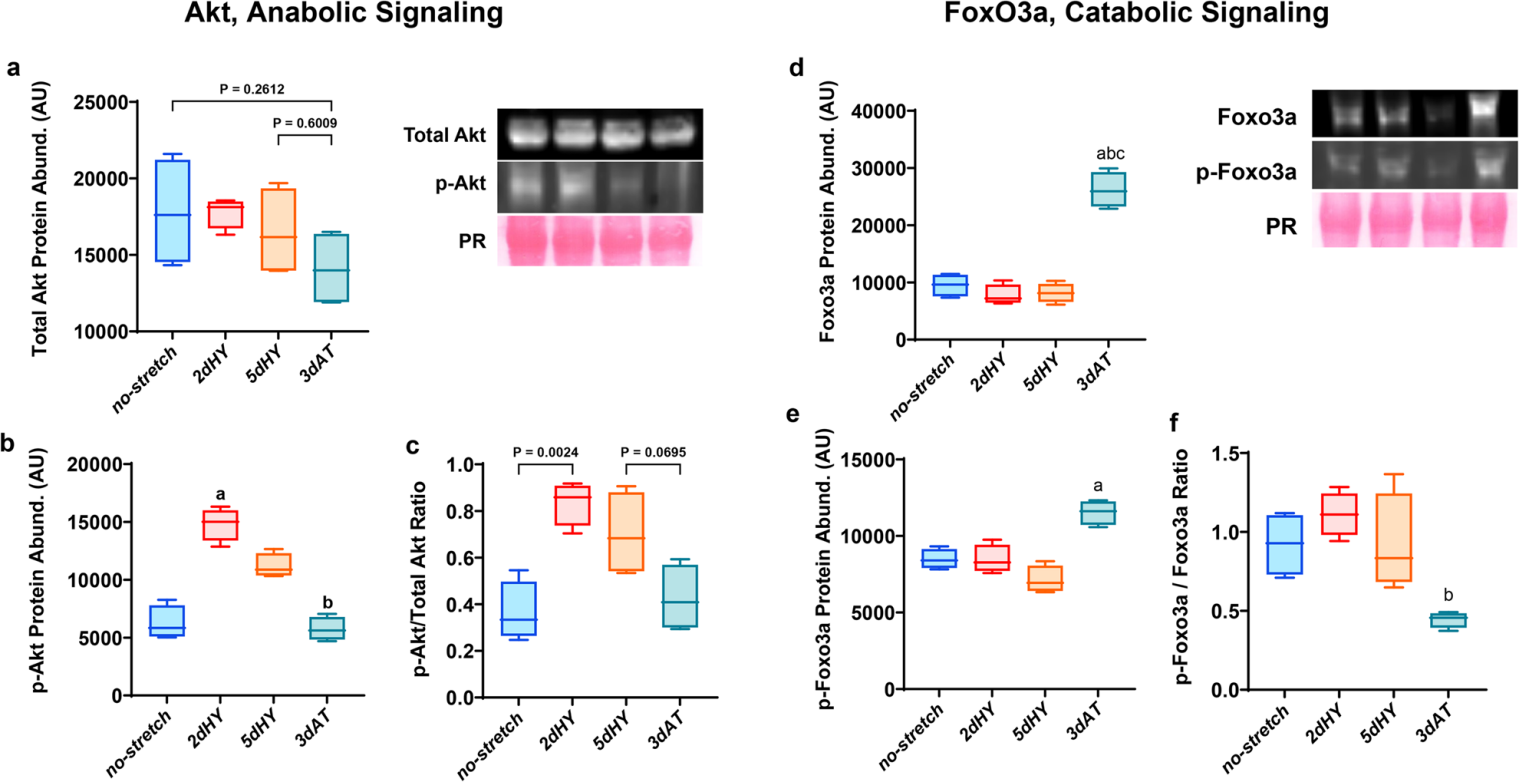
Levels of Akt, anabolic signaling and levels of FoxO3a, catabolic signaling. **a** Total Akt activity. **b** Phosphorylation level of Akt (Thr308). **c** Ratio between phosphorylation Akt (Thr32) and total Akt levels. **d** Total FOXO3a activity. **e** Phosphorylation level of FoxO3a. **f** Ratio between phosphorylation FoxO3a and total FoxO3a levels. Protein abundance quantification was determined using Western immunoblotting with Ponceau stain as a protein loading control and quantified using ImageJ. Myotubes were divided into the following groups (*n* = 4/group): 2 days quiescent control (no stretch (2d), 2 days cyclic stretch (2dHY), 5 days cyclic stretch (5dHY), and 2-days of cyclic stretch followed by a 3-day cessation of stretch (3dAT). (**a**) = statistically significant difference from no-stretch (2d); (**b**) = significantly different than 2dHY. (*p* ≤ 0.05) Values are presented as Box plots ±SD. The boxplots illustrate the interquartile range (25th–75th percentiles) displayed with whiskers spanning from the 10th to the 90th percentiles. The median of the data is indicated by the central line.

**Fig. 4:**
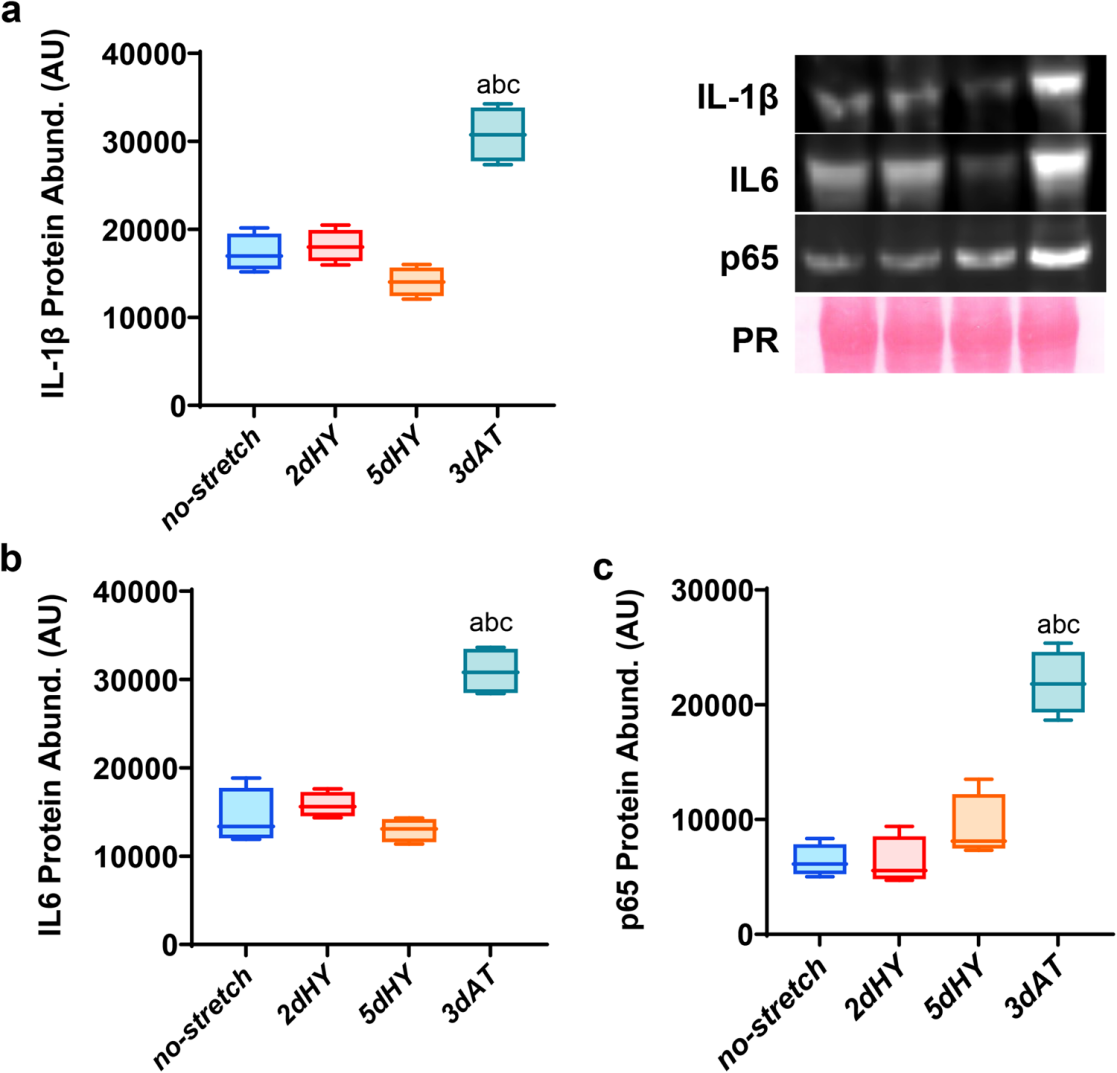
Levels of catabolic, pro-inflammatory markers. **a** Interleukin-1ß (IL-1ß) levels. **b** Interleukin-6 (IL-6) levels. **c** p65 subunit of the pro-inflammatory transcription factor nuclear factor kappa-B (NF-kB). Protein abundance quantification was determined using Western immunoblotting with Ponceau stain as a protein loading control and quantified using ImageJ. Myotubes were divided into the following groups (*n* = 4/group): 2 days quiescent control (no stretch (2d), 2 days cyclic stretch (2dHY), 5 days cyclic stretch (5dHY), and 2-days of cyclic stretch followed by a 3-day cessation of stretch (3dAT). (**a**) = statistically significant difference from no-stretch (2d); (**b**) = significantly different than 2dHY; (**c**) = significantly different than 5dHY. (*p* ≤ 0.05) Values are presented as Box plots ±SD. The boxplots illustrate the interquartile range (25th–75th percentiles) displayed with whiskers spanning from the 10th to the 90th percentiles. The median of the data is indicated by the central line.

The Akt activation pathway has been involved in regulating muscle hypertrophy and atrophy. To reveal the effects of daily cyclic stretch and subsequent cessation of daily bouts of cyclic stretch, we measured total Akt, phosphorylated Akt at Thr308, total protein levels for FoxO3a, phosphorylated FoxO3a, and the p-FoxO3a/total FoxO3a ratio (Fig. 3). Our data revealed that phosphorylated Akt levels were elevated after daily bouts of cyclic stretch (Fig. 4b). The phosphorylated Akt to total Akt activity ratio trended upwards with 2 days of a daily cyclic stretch but was not different from than controls (Fig. 3c). Akt phosphorylation levels were significantly lower in 3dAT than in 2dHY but were no different than controls (Fig. 3b). pAkt/total Akt for 3dAT was similar to the no stretch control group.

The FoxO3a transcription factor can be phosphorylated and inactivated by Akt phosphorylation or conversely dephosphorylated and stimulated by pro-inflammatory signaling. Total protein levels for FoxO3a were elevated by almost 3-fold after cessation of daily cyclic stretch compared with the no stretch, 2dHY and 5dHY groups (Fig. 3d). However, the p-FoxO3a/total FoxO3a levels were significantly lower in the 3dAT group than in controls and trended lower than in the myotubes subjected to daily bouts of cyclic strain (Fig. 3f). The data suggest that FoxO3a levels are elevated, and the proportion of dephosphorylated FoxO3a and thus activated FoxO3a is upregulated with when daily bouts of cyclic stretch are removed.

### Catabolic, inflammatory signaling in response to daily cyclic stretch and cessation of stretch

In order to understand whether the cessation of cyclic strain and subsequent atrophy was linked with catabolic, inflammatory signaling, we interrogated three canonical inflammatory proteins that contribute to skeletal muscle fiber atrophy during unloading. For this purpose, we measured myotube levels of the inflammatory cytokines interleukin-1ß (IL-1ß) and interleukin-6 (IL-6), as well as the p65 subunit of the pro-inflammatory, pro-oxidant transcription factor nuclear factor kappa-B (NF-κB). Protein abundance detected through immunoblotting revealed no statistical difference in IL-1ß, IL-6, and p65 among myotubes subjected to no stretch, 2 days of cyclic stretch, and 5 days of cyclic stretch (Fig. 4). However, there was a 2–3 fold increase in IL-1ß, IL-6, and p65 compared with 2dHY, 5dHY, and the no stretch controls, similar to the pattern observed in total FoxO3a levels. These data suggest a significant role of key pro-inflammatory cytokines and NF-kappaB in myotube atrophy following the removal of daily bouts of cyclic stretch.

## Discussion

In the current study, we examined the efficacy of the application and withdrawal of uniaxial cyclic stretch on myotubes as a prospective bioreactor for use in spaceflight research. The uniaxial cyclic stretch produced tensile loading on myotubes with some shear in anchoring interface with laminin on the dish or membrane. Withdrawal from cyclic stretch elicits an ‘off-transient’ period that our data suggest mimics unloading-microgravity. The cyclic stretch protocol (12% sinusoidal stretch, 0.7 Hz waveform frequency, 1 hour/day) over 2 and 5 days produced marked hypertrophy of skeletal muscle myotubes, accompanied by elevated anabolic markers (talin, Akt phosphorylation at Thr308). In contrast, withdrawal and cessation of cyclic stretch resulted in substantial atrophy on myotube diameter, concomitant with reduced Akt phosphorylation, and elevation of catabolic, pro-inflammatory markers (e.g., IL-1ß, IL-6, NF-kappaB subunit p65) as well as proteolytic signaling (e.g., reduced FoxO3a phosphorylation/total FoxO3a ratio, elevated total FoxO3a). A discussion of the physiological and technological development relevance follows.

These findings have a number of parallels with the data published by Soltow, et al. (2013)^35^ using the FlexCell® as a model for overloading and unloading (e.g., microgravity). The FlexCell®’s 2 days cyclic stretching protocol (12%, 0.7 Hz,1 h/d) using collagen-coated plates increased myotube diameter, talin levels, Akt phosphorylation activity, and FoxO3a phosphorylation leading to stretch-induced muscle hypertrophy and growth. In contrast, 48 h after the removal of bouts of cyclic stretch resulted in decreased anabolic signaling, increased FoxO3a dephosphorylation, thus activating pro-inflammatory and muscle atrophic signaling and causing smaller myotube diameter. These data suggest that uniaxial stretch and cessation of stretch can be validly used to test myotube responses to both increased and decreased mechanical loading.

In contrast, rotating wall vessels bioreactors are commonly used in tissue engineering and cell culture research to simulate microgravity conditions. These bioreactors create a three-dimensional culture environment with low shear forces, mimicking some aspects of weightlessness. However, they are not effective in inducing the primary consequence of weightlessness on skeletal muscles, which is a dramatically reduced tensile load. Furthermore, centrifugation adds compressive stress to alleviate microgravity, a far different loading profile that skeletal muscles and attached tendons experience (e.g., tension along the long axis) in a terrestrial environment.

Increased tensile stress and strain in skeletal muscle fibers promote muscle growth by elevating protein synthesis and altering protein degradation, thus initiating muscle hypertrophy^40–42^. Recent reports also characterized an increase in myotube diameter ex vivo in response to mechanical stretch and stimulation^35,43,44^. In our StrexCell application, the uniaxial cyclic stretching protocol for 2 days and 5 days produced tensile loading on myotubes, which resulted in muscle fiber hypertrophy. Thus, loading of tensile force stimulated an increased myotube diameter and size, confirming that cyclic stretch in myotubes elicits hypertrophy of the muscle fibers (Fig. 1). Overloading produced by cyclic stretch induced muscle hypertrophy that was directly linked to increased markers of anabolism and hypertrophy, including upregulation of talin and increased Akt phosphorylation at Thr308. Akt activation is a causal regulator in protein synthesis signaling in skeletal muscle^45,46^. For example, the overexpression of active Akt levels previously elicited skeletal muscle myotube hypertrophy^47^. Furthermore, we found that bouts cyclic stretch activated Akt through phosphorylation at Thr308, which was linked to an elevated phosphorylated FoxO3a/total FoxO3a ratio which could suppress catabolism. Consistently, we previously demonstrated that the antioxidant enzyme mimetic EUK-134 relieved unloading induced decrease in Akt phosphorylation (deactivation) and decrease in FoxO3a phosphorylation (activation)^5^.

Withdrawal and cessation of the cyclic stretching protocol affect skeletal muscle myotubes producing mechanical unloading alterations. Using the StrexCell system to release from daily cyclic stretch, C2C12 myotubes underwent a significant reduction in myotube size after 3 days cessation of cyclic stretch (Fig. 1). A rapid reduction in skeletal muscle size mimics the muscle disuse atrophy that occurs with mechanical unloading^4,6,35^. Indeed, mechanical unloading and spaceflight lead to both a suppression of anabolic signaling and an elevation of proteolytic pathways, thus leading to muscle fiber atrophy^1,12^. FoxO3a is an atrophy-associated transcription factor that is translocated to the nucleus in its active form during skeletal muscle unloading. Indeed, nuclear accumulation of active FoxO3a is the atrophy-associated transcription factor FoxO3a is linked to muscle atrophy^48,49^. In our StrexCell protocol, withdrawal of daily cyclic stretching led to increased levels of unphosphorylated (i.e., active) FoxO3a, consistent with protein degradation’s role in muscle atrophy (Fig. 3). The total amount of FoxO3a was nearly tripled, also suggesting potential stimulation of catabolic signaling. There was a smaller 40% increase in phosphorylated Foxo3a. thus was a statistically significant decrease (more than 50%) in the p-FoxO3a/total FoxO3a ratio, consistent with greater potential for rapid protein degradation and myotube fiber atrophy. Notably, spaceflight microgravity lowered the Akt activity pathway by downregulating the eIF4/p70S6K signaling^50^. Furthermore, Bodine and Baehr (2014)^51^ reported that the FoxO3a transcription factor regulates the MuRF1 and MAFbx expression involved in the protein degradation process leading to muscle atrophy^52^.

Akt activation is a well-known causal regulator of protein synthesis signaling in skeletal muscle^45,46^. Downregulation of the Akt activation pathway, which is associated with dephosphorylation of Akt and 4E-BP1, has been consistently reported in muscle atrophy^53–55^. Our laboratory previously reported downregulation of Akt and mTOR phosphorylation in response the mechanical unloading, prevented by EUK-134 an antioxidant mimetic of superoxide dismutase and catalase^8^. In this study, Akt phosphorylation was significantly decreased when daily cyclic stretch was withdrawn for three days. This observation would be consistent with reduced protein synthesis and thus muscle atrophy.

Simultaneous activation of FoxO3a and suppression of an Akt-mTOR pathways with skeletal muscle wasting, including mechanical unloading has been a frequent observation^12,40,46,56–58^. Indeed, historical and recent studies have consistently documented increased proteolysis^59^ coupled with decreased skeletal muscle protein synthesis in response to hindlimb unloading of hindlimb muscles^13,60^. Furthermore, we previously documented greater dephosphorylation of FoxO3a, accompanied by downregulation of an Akt-mTOR pathway with unloading-induced skeletal muscle atrophy^8^. This is consistent with the argument that dephosphorylation of Akt can activate FoxO3a and thus enhance proteolysis during unloading-induced atrophy^8,61^. Integration of Akt1 and FoxO3a signaling illustrated the role of Akt in simultaneously regulating muscle protein synthesis and protein degradation^62^.

Various pro-inflammatory signaling pathways activated by cytokines, inflammatory transcription factors, and inflammatory substrates have been implicated in muscle atrophy by affecting muscle protein turnover or myonuclear turnover^63,64^. Unloading-induced muscle atrophy has been consistently linked to an elevation of inflammatory cytokines including interleukin-1-beta (IL-1ß), interleukin-6 (IL-6), interferon-gamma (IFN-γ), and tumor necrosis factor (TNF-α)^65^. Furthermore, the transcription factor NF-kappaB (NF-κB) is often elevated during mechanical unloading and may contribute to the atrophy process^12^. We found that the inflammatory cytokines IL-1ß and IL-6 and the p65 subunit of NF-kappaB were markedly higher in the muscle myotubes after daily cyclic stretching had been discontinued for three days (Fig. 4). The large magnitude of upregulation for IL-2, IL-6, and p65 while myotubes were in a humoral free medium clearly indicated that circulating cytokines and endocrine factors could not exclusively account for the marked elevation of inflammatory signaling. Indeed, these data support the hypothesis that much of inflammatory medication in unloaded muscle fibers could be localized within the muscle fibers. Therefore, upregulation of inflammatory mediators during the loading period must be a result of mechanosensing within the myotubes, rather than circulatory factors.

Physiologically, a 2–3 fold elevation of IL-1ß and IL-6 would be expected to stimulate pro-inflammatory signaling, oxidative stress, protein degradation, and muscle atrophy^66^. Spaceflight and hindlimb unloading-induced atrophy are not surprisingly characterized by increased levels of IL-1ß and IL-6^67,68^. Given that IL-1ß and IL-6 can activate or be activated by NF-κB^57^, thus it was expected that NF-kappaB subunit p65 would also upregulated following cessation of daily cyclic stretch (Fig. 4C). NF-κB has previously found to be involved in mechanical unloading-induced muscle atrophy^69–71^. Upregulation of NF-κB, IL-1ß, and IL-6 can lead to a decrease in phosphorylation of Akt and FoxO3 phosphorylation, consistent with a shift toward catabolic signaling^72^. Therefore, it is likely that the large elevation of IL-1ß, IL-5, and p65 contributed to suppression of anabolism, elevation of catabolic signaling, and thus atrophy of the myotubes.

This study has some limitations, while the unloaded/microgravity model was not free floating we argue that there is a tradeoff is being able to induce cyclic tensile strain and release of cyclic stretch bouts as a reasonable simulation of the unloading of microgravity. Skeletal muscles are complex tissues that generate tensile forces to produce body movement and stability. Muscles adapt to changes in tensile loading by adjusting their mass and cross-sectional area. When mechanical tensile loading is diminished or absent in vivo (e.g., spaceflight, casting, bedrest) during periods of immobility or inactivity, muscle fiber cross-sectional area is decreased in response. Even with the limitations outlined above, myotubes in this study responded significantly to bouts of cycle stretch by myotube hypertrophy and underwent atrophy when unloaded. Thus, our StrexCell model of increased and release of tensile loading resulted in biological responses that would be predictable given the physiological responses to overloading and unloading in vivo.

While tensile loading produces stretch on the myotubes, there is a limitation of a lack of contractile activity during stretch. Indeed, StrexCell modules may not fully mimic resistive training bouts, and there could be significant differences in mechanosensing, Ca^2+^ homeostasis, etc. the response of calpains to both loading and unloading recapitulate in vivo responses to exercise and disuse (Hyatt & Powers 2020). In addition, anabolic, catabolic, and inflammatory responses would be predictable and consistent with in vivo loading and unloading data.

While calpain activity is accepted as an important indicator of cellular Ca2+ overload, we were unable to measure cellular Ca^2+^ directly, using a fluorescence probe (e.g., fluo-4), We will measure [Ca^2+^] levels in future as well as additional, important markers of Ca^2+^ overload such as SERCA1, SERCA2a, sarcolipin, etc.

In conclusion the development of a uniaxial bioreactor for skeletal muscle will assist future studies, on the ground and during spaceflight, to elucidate mechanisms that cause skeletal muscle atrophy and remodeling during spaceflight. In addition, the StrexCell bioreactor would allow direct testing of countermeasure strategies against the adverse effects of spaceflight, including microgravity and radiation. In this study, we tested and developed an alternative bioreactor to mimic the effects of both overloading and microgravity-induced skeletal muscle atrophy by imposing and withdrawing periods of cyclic tensile strain, using a StrexCell uniaxial stretch system. The withdrawal of cyclic stretch was used to demonstrate that mechanical inactivity turns off signaling for muscle hypertrophy, while elevating pro-inflammatory, pro-catabolic pathways that drive protein degradation, thus causing myotube atrophy. Therefore, we conclude that the StrexCell uniaxial stretch system is a viable alternative bioreactor to study spaceflight and re- (over)loading in skeletal muscle cells.

## Methods

### Cell culture proliferation and differential protocol

We used a commonly used immortal cell line (C2C12) (American Type Culture Collection; Manassas, VA, USA) derived from mouse skeletal muscle, generously provide by Dr. James Fluckey’s Muscle Biology Laboratory at Texas A&M University. C2C12 cells were capable of differentiation and are a widely used model to study differentiated skeletal muscle cells. The C2C12 myoblasts were cultured in a 10 cm2 chamber filled with Dulbecco’s modified Eagle’s medium, growth medium (DMEM + NA Pyruvate), supplemented with 10% fetal bovine serum (FBS) and 1% penicillin/streptomycin at 37 °C and 5% CO_2_^35,73^_._ Once the myoblast cultures reached 60–65% confluency, the myoblasts were then trypsinized and re-cultured in 4 well-2 cm^2^ silicone chambers coated by laminin at 37 °C and 5% CO_2_, using 1 ml per well. Once the cells reached 70–80% confluency, myotube differentiation was initiated by switching to differentiation medium DMEM (-) NA Pyruvate, supplemented with 2% horse serum, 1% penicillin/streptomycin, and 0.4% insulin-transferrin-selenium. To study the effects of increased and decreased mechanical stress in C2C12 myotubes, we maintained myotubes on laminin-coated silastic membrane and initiated stretch through a StrexCell® system, then observed the effects of stretch and cessation of stretch on cellular responses^34^. Twelve hours after beginning differentiation, the myotube silicone chambers were exposed to the uniaxial stretching using the StrexCell® unit. The differentiation medium was refreshed daily under sterilized conditions.

### StrexCell loading and unloading protocol

Cyclic stretch of skeletal muscle cell cultures or “myotubes” has been used to mimic the loading pattern of skeletal muscle-induced hypertrophy and atrophy^74^. However, several limitations for the FlexCell system that could impair compatibility for research in spaceflight, including the necessity of a powerful vacuum pump, considerable weight, and difficulty generating true uniaxial stretch. StrexCell® uses a stepper motor and modular contractile cartridges to generate uniaxial stretch on muscle fibers mounted on the silastic membrane. StrexCell is automated and computer-controlled, which enables precise control of the strain levels on the cells, providing a consistent stretch at high and low frequencies of 1–20%.

Myoblasts and myotubes on a StrexCell system were exposed to uniaxial stress/tensile loads and release of tensile loading. While the myotubes were not free floating, they are plated on laminin that simulates the extracellular matrix/endothelium normally attached to muscle fibers surrounding myocytes. The myoblast and myotubes were then suspended in an aqueous medium, which induces equal pressure on all sides. At the micron level, gravity is a relatively weak force compared with adhesions, ion affinity, intermolecular forces, and fluid dynamics, especially in an aqueous medium that surrounds the myoblasts and myotubes. Importantly, adhesion to laminin allows controlled levels of uniaxial stretch to be exercise on myotubes that simulates tensile loading in an aqueous environment.

**Fig. 5:**
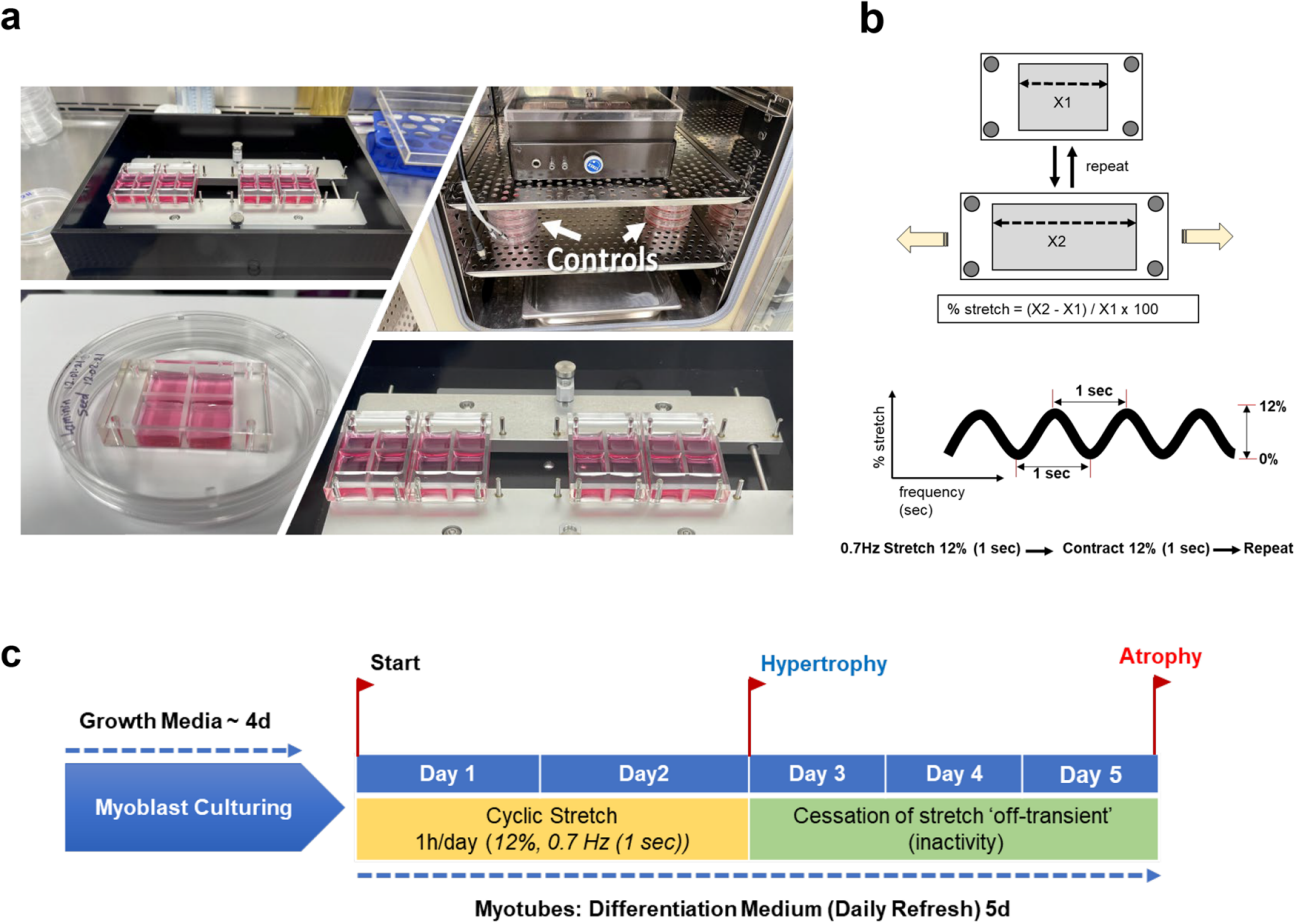
StrexCell proof of concept testing experimental design. **a** STB-1400-10 model: Automated Cell uniaxial stretching system including silicone chamber ((STB-CH-10) 10 cm² stretch chambers, C2C12 myotubes in growth medium), and the static controls (chambers outside the system). **b** Cyclic stretch and cessation protocol timeline and sampling periods. **c** Strain programs rage between 1–20% at frequencies between 1–60 cycle/min using a sine wave pattern.

We used a StrexCell® STB-1400 (B-Bridge International, Inc., Cupertino, CA, USA) to apply a cyclic uniaxial stretch on C2C12 myotubes (Fig. 5a). The stretch instrument employs a computer-controlled stepper motor to drive two metal frames apart. A flexible silicone chamber was attached to both frames, and the chamber was stretched and released in a programmable sinusoidal oscillation. STB-1400 includes a 10 cm^2^ stretch chamber model (Culture area: 3.2 × 3.2 × 1.0 cm), which supports up to 6 chambers simultaneously. The C2C12 cells (2 × 10^5^ cells) were seeded in silicon stretch chambers coated with laminin (Fig. 5a). The amount of strain experienced by the myotubes was measured as the percent change in the stretch from the starting position (Fig. 5b). Based on the preliminary validation experiments and the published report of Soltow, et al. (2013)^35^, the strain program was set at 0.7 Hz frequency, 12% stretch using a sinusoidal wave pattern for 1 hour/day for 2 or 5 days. Exposure to the uniaxial cyclic stretching was utilized to induce hypertrophy with increased loading. Conversely, cyclic stretch cessation was deemed off-transient unloading to induce myotube atrophy release of cyclic tensile loads, with replicate control cultures maintained under quiescent conditions with no applied cyclic strain.

Twenty-four-hour-old myotubes were used at the commencement of this experiment. Five different groups were assigned (*n* = 4/group): (a) 2 days no stretch controls (no Stretch 2d), (b) 5 days no Stretch controls (no Stretch 5d), (c) 2 days cyclic stretching (1 h/d) (2dHY), (d) 5 days cyclic stretching (1 h/d) (5dHY), (e) 2 days cyclic stretching (1 h/d) + 3 days cessation of stretching (3dAT). Myotubes cultures were collected at the end of each group protocol to be fixed for immunofluorescence imaging or homogenized using lysis buffer for western blot analysis.

### Immunofluorescence and confocal microscopy

Immunofluorescence imaging was performed to observe cell morphology. C2C12 myotubes were fixed in a 4% paraformaldehyde (PFA) solution for 30 min at 4 °C. Then, the myotubes were washed three times with 1X PBS. Cell membranes were solubilized for intracellular assays using a 0.5% Triton-X-100 for 3 min until washed with 1X PBS. Myotubes were blocked with 1X PBS with 0.05% Tween20 and 5% goat serum for 30 min at RT. Myotubes were then incubated with β-sarcoglycan (membrane marker) primary antibody (1:200 β-sarcoglycan mouse mAb, abcam, Cat # ab55683) for 1 h at RT, and subsequently washed with 1X PBS (3 × 5 min). Myotubes were then incubated with the appropriate secondary antibody (1:250 goat anti-mouse IgG, Alexa Flour 488, ThermoFisher, Cat # A-11029) for 1 h at RT, followed by washing with 1X PBS (3 × 5 min) and then counterstaining with 1 µg/µl DAPI. After washing with 1X PBS (3 × 5 min) and then double-distilled water (2 × 5 min), slides were mounted with antifade mounting medium (ProLong™ Gold Antifade, Invitrogen, cat # P36930). Myotubes immunofluorescence staining was visualized using a Leica SP8 confocal laser scanning microscope (Leica SP8 Confocal Lightning GPU-based Deconvolution (20X, zoom 2.25, emission wavelength 450–500 nm) in the Texas A&M Image Analysis Core Laboratory. Images were analyzed using Leica LAS X software *v*.4.3 and NIH ImageJ (Fiji. ImageJ *v*.1.53k) software for fiber diameter assessment and nucleus quantification.

### Western immunoblot

Protein abundance was as described by Lawler et al. (2021)^4^ and determined by Western immunoblot analysis. The whole-cell lysate was collected using TRIS HCL lysis buffer for 30 min at 4 °C. The protein concentration was measured using Bradford assay using a microplate reader (Beckman Coulter DTX 880 Multimode Detector). Twenty µg of protein samples were loaded on SDS-PAGE gels and electrophoresed at 120 V for 75 min using a Bio-Rad Protein III gel box onto a nitrocellulose membrane (Bio-Rad: Hercules, CA). The membranes were then blocked in a non-fat milk buffer (5% non-fat milk in TBS) for 1 h. Following blocking, membranes were incubated overnight (4 °C) in a blocking buffer with the appropriate primary antibodies: Anabolic signaling (anti-total Akt (rabbit mAb; 1:500; Cell Signaling Cat# 4691 S), anti-Akt phosphorylation at Thr308 (rabbit pAb; 1:500; Cell Signaling Cat# 9271 S), anti-Talin (rabbit mAb; 1:500; Cell Signaling Cat# 4021); Catabolic signaling with anti-Foxo3a (rabbit mAb; 1:500; Cell Signaling Cat# 12829 S) and anti-phospho-Foxo3a at Ser473 (rabbit pAb; 1:300; Cell Signaling Cat# 9464 L); inflammatory markers anti-IL-1β (rabbit mAb; 1:1,000; Cell Signaling, Cat #12703), anti-IL-6 (rabbit mAb; 1:1,000; Cell Signaling, Cat #12912), and anti-P65 (mouse mAb; 1:500; abcam, Cat# ab32536). Then, the membranes were washed with T-TBS (3 × 5 min) and incubated with the secondary antibody for 1 h at 4 °C.

Proteins were visualized using Clarity Western ECL Substrate (BioRad, cat# 1705061) enhanced chemiluminescence detection and developed using a Cytiva Amersham ImageQuant™ 800 Western Blot Imaging System (Cytiva). Quantification was performed by ImageQuant™ TL10 *v*.10.2 and NIH ImageJ software with Ponceau-S staining serving as a loading control.

### Calpain activity

Calpain activity was performed using a Calpain Activity Assay Fluorometric Kit (Abcam # ab65308) according to the manufacturer’s instructions, using 20 µg of the whole-cell lysate (mixed with lysis buffer provided with the kit). Calpain activities for each sample were expressed as relative fluorescence units (RFUs) per milligram of protein per minute (RFU/μg/min). The fluorescence signal was detected at 400 nm excitation and 505 nm emission using a microplate reader (Beckman Coulter DTX 880 Multimode Detector).

### Statistical approach

Results were collected and processed for statistical analysis using Prism 9.0 (GraphPad). One-way ANOVAs with Tukey’s post-hoc analysis was performed to distinguish among group mean differences and between the different variables with significance set at *P* < 0.05. A nested ANOVA design was used to quantify myotube diameter.

### Reporting summary

Further information on research design is available in the Nature Research Reporting Summary linked to this article.

### Supplementary information

Reporting Summary

## Data Availability

All data generated or analyzed during this study are included in this published article.
