# Supplementary material for: Bioreactor development for skeletal muscle hypertrophy and atrophy by manipulating uniaxial cyclic strain: proof of concept
Source: NPJ Microgravity. 2024 Jun 11;10:62. doi: 10.1038/s41526-023-00320-0 (PMC11167039; doi:10.1038/s41526-023-00320-0)
Supplement: Supplementary file 1 — Reporting Summary [file 41526_2023_320_MOESM1_ESM.pdf]

nature portfolio

Corresponding author(s): Khaled Y. Kamal

Last updated by author(s): 07/11/2023

Reporting Summary

Nature Portfolio wishes to improve the reproducibility of the work that we publish. This form provides structure for consistency and transparency in reporting. For further information on Nature Portfolio policies, see our Editorial Policies and the Editorial Policy Checklist.

Statistics

For all statistical analyses, confirm that the following items are present in the figure legend, table legend, main text, or Methods section.

n/a	Confirmed	
☐	☒	The exact sample size (*n*) for each experimental group/condition, given as a discrete number and unit of measurement
☐	☒	A statement on whether measurements were taken from distinct samples or whether the same sample was measured repeatedly
☐	☒	The statistical test(s) used AND whether they are one- or two-sided<br>*Only common tests should be described solely by name; describe more complex techniques in the Methods section.*
☒	☐	A description of all covariates tested
☒	☐	A description of any assumptions or corrections, such as tests of normality and adjustment for multiple comparisons
☒	☐	A full description of the statistical parameters including central tendency (e.g. means) or other basic estimates (e.g. regression coefficient) AND variation (e.g. standard deviation) or associated estimates of uncertainty (e.g. confidence intervals)
☒	☐	For null hypothesis testing, the test statistic (e.g. *F*, *t*, *r*) with confidence intervals, effect sizes, degrees of freedom and *P* value noted<br>*Give P values as exact values whenever suitable.*
☒	☐	For Bayesian analysis, information on the choice of priors and Markov chain Monte Carlo settings
☒	☐	For hierarchical and complex designs, identification of the appropriate level for tests and full reporting of outcomes
☒	☐	Estimates of effect sizes (e.g. Cohen's *d*, Pearson's *r*), indicating how they were calculated

Our web collection on statistics for biologists contains articles on many of the points above.

Software and code

Policy information about availability of computer code

Data collection	Myotubes immunofluorescence staining was visualized using a Leica SP8 confocal laser scanning microscope (Leica SP8 Confocal Lightning GPU-based Deconvolution (20X, zoom 2.25, emission wavelength 450-500 nm) in the Texas A&M Image Analysis Core Laboratory. Images were collected using Leica LAS X software v.4.3
Data analysis	1- Confocal Images were were analyzed using Leica LAS X software v.4.3 and NIH ImageJ (Fiji. ImageJ v.1.53k) software for fiber diameter assessment and nucleus quantification.<br>2- Western Blot (protein levels) quantification was performed by ImageQuant™ TL10 v.10.2 and NIH ImageJ software.<br>3- Statistical analysis were collected and processed for statistical analysis using Prism 9.0 (GraphPad).

For manuscripts utilizing custom algorithms or software that are central to the research but not yet described in published literature, software must be made available to editors and reviewers. We strongly encourage code deposition in a community repository (e.g. GitHub). See the Nature Portfolio guidelines for submitting code & software for further information.

Data

Policy information about availability of data

All manuscripts must include a data availability statement. This statement should provide the following information, where applicable:

- Accession codes, unique identifiers, or web links for publicly available datasets
- A description of any restrictions on data availability
- For clinical datasets or third party data, please ensure that the statement adheres to our policy

Provide your data availability statement here.

Research involving human participants, their data, or biological material

Policy information about studies with human participants or human data. See also policy information about sex, gender (identity/presentation), and sexual orientation and race, ethnicity and racism.

Reporting on sex and gender	not applicable
Reporting on race, ethnicity, or other socially relevant groupings	not applicable
Population characteristics	not applicable
Recruitment	not applicable
Ethics oversight	not applicable

Note that full information on the approval of the study protocol must also be provided in the manuscript.

Field-specific reporting

Please select the one below that is the best fit for your research. If you are not sure, read the appropriate sections before making your selection.

☒ Life sciences ☐ Behavioural & social sciences ☐ Ecological, evolutionary & environmental sciences

For a reference copy of the document with all sections, see nature.com/documents/nr-reporting-summary-flat.pdf

Life sciences study design

All studies must disclose on these points even when the disclosure is negative.

Sample size	this is in vitro experiments using cell cultures, no sample calculation was performed.
Data exclusions	no data were excluded
Replication	all attempt for replication has been successful
Randomization	samples between the groups and within the groups have been completely randomized
Blinding	*Describe whether the investigators were blinded to group allocation during data collection and/or analysis. If blinding was not possible, describe why OR explain why blinding was not relevant to your study.*

Reporting for specific materials, systems and methods

We require information from authors about some types of materials, experimental systems and methods used in many studies. Here, indicate whether each material, system or method listed is relevant to your study. If you are not sure if a list item applies to your research, read the appropriate section before selecting a response.

Materials & experimental systems

n/a	Involved in the study	
☐	☒	Antibodies
☐	☒	Eukaryotic cell lines
☒	☐	Palaeontology and archaeology
☒	☐	Animals and other organisms
☒	☐	Clinical data
☒	☐	Dual use research of concern
☒	☐	Plants

Methods

n/a	Involved in the study	
☒	☐	ChIP-seq
☒	☐	Flow cytometry
☒	☐	MRI-based neuroimaging

Antibodies

Antibodies used	Anabolic signaling (anti-total Akt (rabbit mAb; 1:500; Cell Signaling Cat# 4691S), anti-Akt phosphorylation at Thr308 (rabbit pAb; 1:500; Cell Signaling Cat# 9271S), anti-Talin (rabbit mAb; 1:500; Cell Signaling Cat# 4021); Catabolic signaling with anti-Foxo3a (rabbit mAb; 1:500; Cell Signaling Cat# 12829S) and anti-phospho-Foxo3a at Ser473 (rabbit pAb; 1:300; Cell Signaling Cat# 9464L); inflammatory markers anti-IL-1β (rabbit mAb; 1:1,000; Cell Signaling, Cat #12703), anti-IL-6 (rabbit mAb; 1:1,000; Cell Signaling, Cat #12912), and anti-P65 (mouse mAb; 1:500; abcam, Cat# ab32536).
Validation	all these antibodies are commercial, and all the citations, and validation are available in the manufacture website.

Eukaryotic cell lines

Policy information about cell lines and Sex and Gender in Research

Cell line source(s)	We used a commonly used immortal cell line (C2C12) derived from mouse skeletal muscle, generously provide by Dr. James Fluckey's Muscle Biology Laboratory at Texas A&M University. C2C12 cells were capable of differentiation and are a widely used model to study differentiated skeletal muscle cells
Authentication	non of the cell line was authenticated
Mycoplasma contamination	All the cell line were tested negative for the mycoplasma contamination.
Commonly misidentified lines (See ICLAC register)	N/A
